# A Yeast Model of FUS/TLS-Dependent Cytotoxicity

**DOI:** 10.1371/journal.pbio.1001052

**Published:** 2011-04-26

**Authors:** Shulin Ju, Daniel F. Tardiff, Haesun Han, Kanneganti Divya, Quan Zhong, Lynne E. Maquat, Daryl A. Bosco, Lawrence J. Hayward, Robert H. Brown, Susan Lindquist, Dagmar Ringe, Gregory A. Petsko

**Affiliations:** 1 Department of Biochemistry and Chemistry, Rosenstiel Basic Medical Sciences Research Center, Brandeis University, Waltham, Massachusetts, United States of America; 2 Department of Neurology and Center for Neurologic Diseases, Harvard Medical School and Brigham & Women's Hospital, Cambridge, Massachusetts, United States of America; 3 Whitehead Institute for Biomedical Research, Cambridge, Massachusetts, United States of America; 4 Howard Hughes Medical Institute, Department of Biology, Massachusetts Institute of Technology, Cambridge, Massachusetts, United States of America; 5 Department of Cancer Biology, Dana Farber Cancer Institute, Boston, Massachusetts, United States of America; 6 Department of Genetics, Harvard Medical School, Boston, Massachusetts, United States of America; 7 Department of Biochemistry and Biophysics and Center for RNA Biology, School of Medicine and Dentistry, University of Rochester, Rochester, New York, United States of America; 8 Department of Neurology, University of Massachusetts Medical School, Worcester, Massachusetts, United States of America; University of California San Francisco/Howard Hughes Medical Institute, United States of America

## Abstract

FUS/TLS is a nucleic acid binding protein that, when mutated, can cause a subset of familial amyotrophic lateral sclerosis (fALS). Although FUS/TLS is normally located predominantly in the nucleus, the pathogenic mutant forms of FUS/TLS traffic to, and form inclusions in, the cytoplasm of affected spinal motor neurons or glia. Here we report a yeast model of human FUS/TLS expression that recapitulates multiple salient features of the pathology of the disease-causing mutant proteins, including nuclear to cytoplasmic translocation, inclusion formation, and cytotoxicity. Protein domain analysis indicates that the carboxyl-terminus of FUS/TLS, where most of the ALS-associated mutations are clustered, is required but not sufficient for the toxicity of the protein. A genome-wide genetic screen using a yeast over-expression library identified five yeast DNA/RNA binding proteins, encoded by the yeast genes *ECM32*, *NAM8*, *SBP1*, *SKO1*, and *VHR1*, that rescue the toxicity of human FUS/TLS without changing its expression level, cytoplasmic translocation, or inclusion formation. Furthermore, *hUPF1*, a human homologue of *ECM32*, also rescues the toxicity of FUS/TLS in this model, validating the yeast model and implicating a possible insufficiency in RNA processing or the RNA quality control machinery in the mechanism of FUS/TLS mediated toxicity. Examination of the effect of FUS/TLS expression on the decay of selected mRNAs in yeast indicates that the nonsense-mediated decay pathway is probably not the major determinant of either toxicity or suppression.

## Author Summary

Of all the thousand natural shocks that flesh is heir to, one of the most devastating is amyotrophic lateral sclerosis (ALS), commonly known as Lou Gehrig's Disease. This disorder, which comes in both inherited and random forms, is characterized by degeneration of spinal motor neurons, leading to paralysis and death. The cause of the sporadic form is unknown, but new insight has come from studying the genetic variations that lead to the rarer familial forms. One such gene, accounting for 5%–10% of inherited ALS, is FUS/TLS, which encodes a protein that normally lives in the nucleus of the cell and is involved in the life-cycle of messenger RNA (mRNA). ALS-associated mutations in FUS/TLS cause the protein to mislocalize outside the nucleus into stress granules. Understanding the basis for the toxicity of mislocalized FUS/TLS could lead to new approaches to the treatment of ALS. We have made a yeast model for FUS/TLS cellular toxicity that recapitulates the mislocalization, granular accumulation, and cell death. We have exploited the yeast model to obtain information about what part of the protein is required for proper localization and what part is essential for toxicity. We have also identified several human genes that, when over-expressed in yeast, are able to rescue the cell from the toxicity of mislocalized FUS/TLS. These genes all have functions in mRNA quality control, implicating changes in this pathway in the pathology of ALS.

## Introduction

Amyotrophic lateral sclerosis (ALS, also called Lou Gehrig's disease after one of its most famous victims) is a relentlessly progressive, fatal neurodegenerative disease with a prevalence of ∼5 people out of 100,000 each year and an average age of onset of ∼60 years. Patients with ALS suffer from degeneration of motor neurons in the brain and spinal cord, which leads to progressive muscular weakness. ALS accounts for ∼1/300 to 1/400 of all deaths, which means that about 1,000,000 people now alive in the United States will develop ALS. Death typically occurs 3–5 years after disease onset, due to respiratory paralysis. There is no effective treatment for the disease; the only approved ALS drug (riluzole) extends the lifespan of some ALS patients by only about 3 months.

While most forms of ALS are sporadic and idiopathic (sALS), ∼10% of cases are inherited in a Mendelian fashion and are designated familial ALS (fALS). As is the case for Parkinson's and Alzheimer's diseases, which also have ∼10% familial forms, genetic analysis has identified several genes that cause fALS. The first mutations were identified in *SOD1*, which encodes the ubiquitously expressed copper/zinc superoxide dismutase; these variants cause ∼20% of fALS worldwide. More than 150 different ALS mutations, spanning virtually the entire coding sequence of the highly conserved *SOD1* gene, have been identified—nearly all of them exhibiting autosomal dominant inheritance [1]. Although inclusions containing aggregated SOD1 protein have been found in the spinal motor neurons of patients with SOD1-dependent fALS, they are generally not found in the sporadic disease.

More recently, other genes have been identified that collectively account for a significant percentage of the remaining fALS cases. These include the genes coding for alsin (ALS2), vesicle associated membrane protein B (VAPB) [2], senataxin (SETX) [3], TAR-DNA-binding protein (TDP-43) [4], fused in sarcoma or translocated in liposarcoma (FUS*/*TLS) [5],[6], and optineurin (OPTN) [7]. A small number of other genes have been associated with increased risk for sALS, most recently ataxin-2 [8]. Studies of these genes have provided important information about the biochemical processes that may underlie ALS. Putative mechanisms of toxicity targeting motor neurons include glutamate excitotoxicity, oxidative damage, proteasome inhibition, mitochondrial dysfunction, ER stress, axonal transport defects, growth factor signaling deficiency, and glial cell dysfunction [9],[10].

Two of the genes associated with fALS, FUS/TLS and TDP-43, are of special interest because inclusions containing these proteins have been identified in motor neurons of both sporadic and familial patients [11]–[15]. In addition, both of these genes have been linked to rare forms of frontotemporal lobar degeneration [16], indicating that they play crucial roles in other neurons. FUS/TLS and TDP-43 are both predominantly nuclear RNA binding proteins, although they have also been reported to bind DNA in vitro. Both FUS/TLS and TDP-43 are believed to carry out important functions in multiple steps of RNA processing, including transcription, splicing, transport, translation, and decay [17]. The finding that both are fALS genes (each accounts for about 5% of familial ALS cases), and are involved in sALS, raises the possibility that RNA processing or quality control (damage repair and decay of prematurely terminated messages) may be central to ALS pathology. However, the precise connections between RNA biology and ALS remain to be discovered.

The FUS/TLS protein, which is ubiquitously expressed in all tissues, contains an N-terminal putative transcriptional activation domain (residues 1–267) rich in serine, tyrosine, glutamine, and glycine residues, followed by a canonical RNA binding domain (residues 285–371). The C-terminal region has a zinc finger domain (residues 422–453) interrupting a long stretch rich in arginines and glycines (residues 285–501). The extreme C-terminal 25 amino acids (residues 501–526) also are rich in arginines and glycines, and the majority of the ALS-associated mutations are found among them. Among other postulated functions, FUS/TLS is known to be a component of a large nuclear ribonucleoprotein complex that functions in shuttling mRNA out of the nucleus.

Variants of FUS/TLS have previously been studied for their role in liposarcoma, in which the N-terminal transcriptional activation domain of FUS/TLS is translocated into another chromosomal locus, resulting in gene fusions and production of chimeric oncoproteins (e.g. FUS-ERG, FUS-CHOP, and FUS-CREB312). The fusion proteins are aberrant transcription factors that contribute to the tumorigenic process by altering the expression of many target genes [18].

Mutations in FUS/TLS found in fALS are largely clustered at the extreme C-terminus of the protein. Postmortem histological analysis from patients with FUS/TLS mutations indicates that the normally nuclear protein is now found more predominantly in the cytosol, where it forms punctate inclusions. This mislocalization/inclusion-formation has been proposed to cause either a loss of normal protein function in the nucleus, a gain of toxic function in the cytosol, or both [5],[17]. Recently, Dormann et al. [19] reported that some of the disease-causing mutations affect a non-classical PY nuclear localization signal (NLS) in the extreme C-terminus of FUS/TLS and disrupt transportin-mediated nuclear import of the protein. As a result, FUS/TLS distribution increases in the cytosol, where the protein can be recruited into stress granules [20]. These results have led to the hypothesis that nuclear import defects and consequent cellular stress may be necessary, and possibly sufficient, for FUS/TLS pathogenesis [19],[20].

Since FUS/TLS-immunoreactive inclusions are reported to be a common feature in both sporadic and familial ALS [13], it is likely that an understanding of FUS/TLS-associated fALS could also provide valuable information about the more common sporadic form of the disease. Additionally, the involvement of FUS/TLS in other neurodegenerative diseases, such as a subset of FTLD (atypical FTLD-U) [21], neuronal intermediate filament inclusion disease (NIFID) [22], and polyglutamine disease [23], suggests that several neurodegenerative diseases may have similar underlying pathogenic mechanisms. A better understanding of the normal and aberrant functions of FUS/TLS might therefore provide clues to uncovering the pathology of neurodegenerative diseases beyond ALS. With this in mind, we set out to create a model for FUS/TLS-dependent cytotoxicity in a genetically and biochemically tractable organism.

With uniquely available genetic and biochemical tools, yeast has proven to be a valuable system to study the functions of human proteins involved in many diseases, including neurodegenerative disorders [24]–[29]. Although yeast is a simple single-cell eukaryote, many fundamental cellular processes are conserved between yeast and higher eukaryotes, and a number were first discovered in *S. cerevisiae* or its distant relative, *S. pombe*. If expression of FUS/TLS in yeast can be shown to recapitulate some of the relevant features of human FUS/TLS-dependent proteotoxicity, then genetic screens can be used to dissect the pathways and processes involved.

In this article, we report a yeast model of FUS/TLS-dependent cytotoxicity, in which over-expression and mislocalization of wild-type or mutant FUS/TLS recapitulates the phenotypes of toxicity and inclusion formation observed in the human disease. Certain features of the model have allowed us to conclude that cytosolic localization of large amounts of even the wild-type FUS protein is sufficient to cause toxicity, supporting a hypothesis that had been put forward based on studies in mammalian cells. A genetic screen using this yeast model for suppressors of toxicity identifies, among other genes, the yeast gene *ECM32*, an RNA helicase involved in RNA quality control, as one that rescues FUS/TLS toxicity when over-expressed. In addition, *hUPF1*, a human homolog of *ECM32*, rescues FUS/TLS toxicity, as do its interacting partners *hUPF2* and (to a lesser extent) *hUPF3*. The rescue does not involve a decrease in FUS/TLS expression or a change in its localization or inclusion formation, but it does depend on intact functional domains of *hUPF1*. Since *hUPF1* plays an important function in mRNA quality control, our data raise the possibility that this pathway might be involved in the pathogenesis of FUS/TLS*-*associated ALS, and possibly of the disease in general. We have investigated the role of one aspect of RNA quality control, nonsense-mediated decay (NMD), using the yeast model but find no evidence that NMD disruption is responsible for FUS/TLS toxicity or that its upregulation is important for suppression.

Independently, Sun et al. (2010, [30]) have developed a similar yeast model for FUS/TLS-associated ALS and have identified all of the same suppressor genes, plus some additional ones, in a similar screen. In addition, they have found genes that, when deleted, modulate FUS/TLS toxicity in yeast, and have implicated stress granules (discrete cytoplasmic phase-dense particles, observed in cells exposed to heat, oxidative, hyperosmolarity, and UV stress, where non-translating mRNAs are stored) in this process.

## Results

### Expression and Localization of Human FUS/TLS in Yeast: Over-expression of FUS/TLS Is Toxic

**Figure 1:**
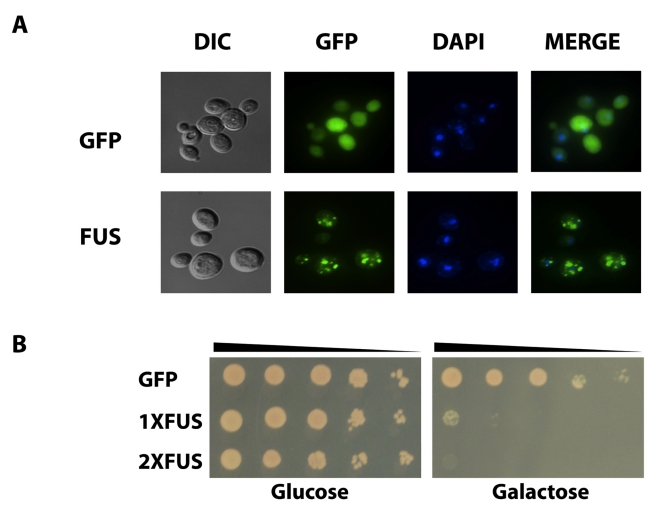
Expression, localization, and toxicity of FUS/TLS in yeast. (A) Cells expressing GFP or GFP-FUS were induced by 2% galactose for 6 h. Cells were then fixed and viewed by fluorescence microscopy. DAPI was used to stain the nucleus. (B) Yeast with integrated GFP, 1XFUS (1 copy of untagged FUS integrated into the *HIS3* locus in the genome), and 2XFUS (2 copies of untagged FUS integrated into the *HIS3* locus and the *TRP1* locus in the genome) were serially diluted (from left to right) and spotted onto plates containing either glucose (FUS expression “off”) or galactose (expression “on”). Picture was taken after 2 d growth at 30°C.

Unlike wild type FUS/TLS, which is largely found in the nucleus and somewhat diffusely in the cytosol, the mutant proteins associated with fALS are predominantly aggregated in the cytoplasm of neurons, where they are proposed to be toxic. To determine whether the budding yeast *S. cerevisiae* may serve as a model for investigating the molecular mechanisms of FUS/TLS cytotoxicity, we generated yeast strains expressing FUS/TLS. The human *FUS/TLS* gene, in both wild type and mutant (R521G and H517Q) forms, was N-terminally fused to green fluorescent protein (GFP) and placed under the control of the *GAL1* promoter, whereby expression is tightly controlled by switching the carbon source in the medium. In these strains, expression of both WT and mutant FUS/TLS is highly induced by shifting to galactose medium. When either mutant or wild-type protein is over-expressed (induced by 2% galactose), the majority of the protein forms punctate aggregates in the cytosol (Figure 1A, and Figure S1), recapitulating the nuclear to cytoplasmic translocation phenotype characteristic of FUS/TLS-associated sporadic and familial ALS, as well as other neurodegenerative diseases [5]. Data from an independent yeast model of FUS/TLS cytoxicity [30] and expression of mutant forms of FUS/TLS in mammalian cells [20] suggest that these aggregates could be localized to stress granules.

To test possible toxicity of aggregated FUS/TLS in the cytosol, one copy of FUS/TLS (1XFUS; untagged) and two copies of FUS/TLS (2XFUS; untagged) were integrated into the genome of yeast strain W303α. Yeast strains with FUS/TLS (1XFUS and 2XFUS) were serially diluted and spotted onto plates with glucose (expression repressed) and galactose (expression induced). As shown in Figure 1B, FUS/TLS over-expression is toxic to yeast in a dose-dependent manner under these proliferative growth conditions, with the 2XFUS exhibiting greater toxicity than 1XFUS. Toxicity of the over-expressed mutant proteins was comparable to that of the wild-type (Figure S1; see below for an explanation), so the wild-type protein was used for most of the remaining experiments, to avoid possible sequence-dependent peculiarities.

### Inclusions Formed by FUS/TLS Are Different from Those Formed by PolyQ-Expanded Huntingtin

In yeast models for several other protein misfolding diseases, although the morphologies of the aggregated proteins under the fluorescence microscope look similar, the actual characteristics of the aggregates are sometimes quite different. For example, yeast toxicity and aggregation of the human Huntingon's disease–associated protein huntingtin harboring a pathogenic polyglutamine expansion (Htt103Q) can be rescued by the deletion of a heat shock protein *HSP104* (*hsp104Δ*) or yeast prion protein RNQ1 (*rnq1Δ*). However, the same deletions cannot rescue the cytosolic aggregation and toxicity of TDP-43 in yeast [28]. Previous studies also indicate that Htt103Q forms SDS-insoluble aggregates, which cannot pass through a 0.2 µM cellulose acetate membrane; however, TDP43 aggregates can pass freely [28].

**Figure 2:**
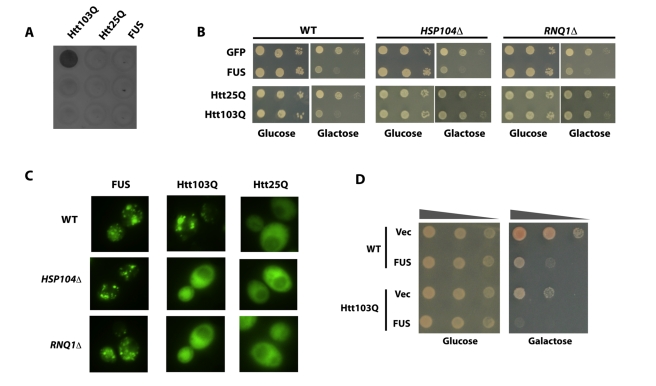
Aggregates of FUS/TLS are different from that of Huntingtin. (A) Yeast cells containing GFP-tagged N-terminal huntingtin harboring pathogenic polyglutamine expansions (Htt103Q, stretch of 103 consecutive glutamines), normal huntingtin (Htt25Q, stretch of 25 consecutive glutamines), and GFP tagged FUS/TLS were induced with galactose for 6 h. Filter retardation assay was performed to characterize the aggregates. (B) GFP tagged FUS, Htt103Q, or Htt25Q was transformed into *HSP104Δ*, *RNQ1Δ* deletion strains and the isogenic wild type strain BY4743 (WT). Cells were serially diluted and spotted onto glucose (expression “off”) or galactose plate (expression “on”) to observe toxicity. Pictures were taken after 2 d growth at 30°C. GFP on the same vector (GFP) was used as control. (C) The same strains as above were visualized for localization and aggregation of the proteins by fluorescence microscopy. (D) pYES2CT/GFP-FUS (FUS) and empty vector (Vec) were transformed into wild type yeast, and yeast containing integrated Htt103Q. Freshly grown cells were then serially diluted and spotted onto glucose (expression “off”) or galactose plate (expression “on”) to observe toxicity. Pictures were taken after 2 d growth at 30°C.

FUS/TLS has been found to be associated with huntingtin aggregates in Huntington's disease patients [23], yet the characteristics of FUS/TLS-dependent fALS resemble those of TDP-43-associated ALS. To test possible differences between FUS/TLS aggregates and huntingtin or TDP-43, FUS/TLS aggregates isolated from over-expressing yeast were tested by a filter retardation assay. As expected, Htt103Q was trapped by the membrane; however, FUS/TLS aggregates, like TDP43, passed through the membrane freely (Figure 2A). Consistent with this observation, unlike their effects on Htt103Q, deletion of *HSP104* and *RNQ1* did not modify the toxicity of FUS/TLS in yeast (Figure 2B), nor its aggregation or localization (Figure 2C). In addition, over-expression of *HSP104* and *RNQ1* had no effect on FUS/TLS toxicity either (unpublished data).

These differences between FUS/TLS and Htt103Q aggregates suggest that the toxicity mechanism underlying these two proteins might be different. Consistent with this, toxicity resulting from the over-expression of FUS/TLS and Htt103Q is additive (Figure 2D).

### The C-Terminal Domain of FUS/TLS Is Necessary But Not Sufficient for Its Toxicity

**Figure 3:**
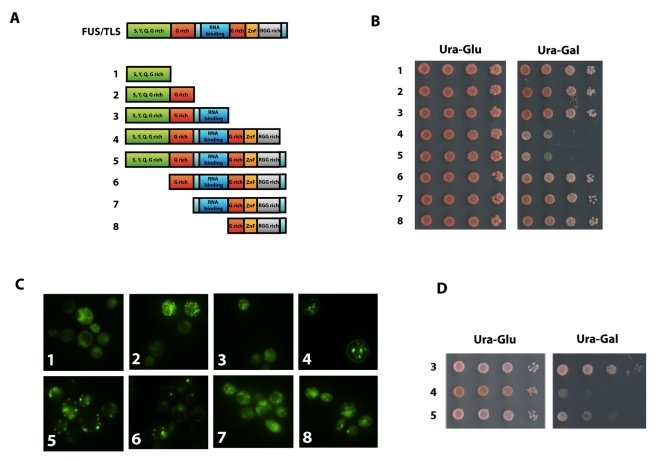
Toxicity and localization of individual domains of FUS/TLS. (A) A serial deletion of the full length FUS/TLS gene from c-terminus, labeled as 1–4, and from N-terminus, labeled as 6–8, was carried out. (B) The truncated genes (with GFP tag at N-terminus) were then placed under the control of *GAL1* promoter on the yeast expression vector pYES2CT. Yeast with above constructs was serially diluted and spotted onto plate containing either glucose (expression “off”) or galactose (expression “on”). Pictures of the plates were taken after 2 d growth at 30°C. (C) Cells containing the above constructs were grown in the Ura-Raffinose medium to mid-log phase. Expression of the proteins was induced by 2% galactose for 6 h. Localization and aggregation of the proteins was visualized by fluorescence microscopy. (D) Constructs 3–5 as shown in (A) were also cloned into yeast expression vector pDEST52 without the GFP tag. Yeast containing the constructs was serially diluted and spotted onto glucose (expression “off”) or galactose plate (expression “on”). Picture of the plates was taken after 2 d growth at 30°C.

The full-length FUS/TLS protein has an N-terminal transcriptional activation domain (residues 1–267) including SYQG-rich (residues 1–164) and G-rich (residues 165–267) subdomains, and a C-terminal RNA binding region (residues 285–526) including RNA binding (residues 285–370), Zinc Finger (residues 422–452), and RGG-rich domains (residues 371–421 and 453–501) (Figure 3A). The extreme C-terminus (residues 511–526) contains an arginine-rich sequence and a putative nuclear localization signal [19]; every one of the arginines in this region is the site of an ALS-associated mutation. To pinpoint which part of FUS/TLS is required for its toxicity in yeast, we characterized eight fragments of the wild-type FUS/TLS protein (tagged with GFP at the N-terminus) for their localization, aggregation, and toxicity. As shown in Figure 3B, after removal of the C-terminal region of the protein (constructs 1 [residues 1–164], 2 [residues 1–267], and 3 [residues 1–370]), the protein is no longer toxic, suggesting that the C-terminal domain from residues 371 on is required for toxicity. However, expression of the C-terminal domain only (constructs 6 [residues 165–526], 7 [residues 268–526], and 8 [residues 371–526]) is not toxic, indicating that both C- and N-terminal regions of the protein are essential for toxicity.

To check for a possible correlation between toxicity and aggregation, all eight proteins (N-terminally GFP tagged) were checked using fluorescence microscopy. As shown in Figure 3C, all the proteins, when over-expressed, show aggregation; however, only constructs 4 (which lacks the extreme C-terminal 15 residues) and 5 (the full-length protein) are toxic. Interestingly, the aggregates formed by constructs 4 and 5 appear slightly different from each other and also may differ from the aggregates formed by the other constructs. These data suggest that toxicity of FUS/TLS in yeast involves mechanisms beyond protein aggregation, a hypothesis supported by the results of the suppressor screen (see below).

To test the possible effects of the GFP tag on toxicity, we expressed constructs 3–5 without the N-terminal GFP fusion. Consistent with non-tagged constructs, construct 3, which lacks the residues from 371 to 526, is not toxic, and constructs 4 and 5 are still toxic. Interestingly, the construct lacking the 15 amino acids at the extreme C-terminal end (construct 4), where most of the fALS mutations are clustered, is more toxic to yeast than the full-length protein (construct 5). This result is consistent with the recent finding that patients with a nonsense mutation of FUS/TLS at position 495 have more rapidly progressive neurodegeneration and earlier onset of the disease [20],[31] and that the C-terminus encodes a nuclear localization signal [19].

### Localization and Toxicity of FUS/TLS in Yeast Is Not Regulated by Either of the Major Yeast Arginine Methyltransferases

**Figure 4:**
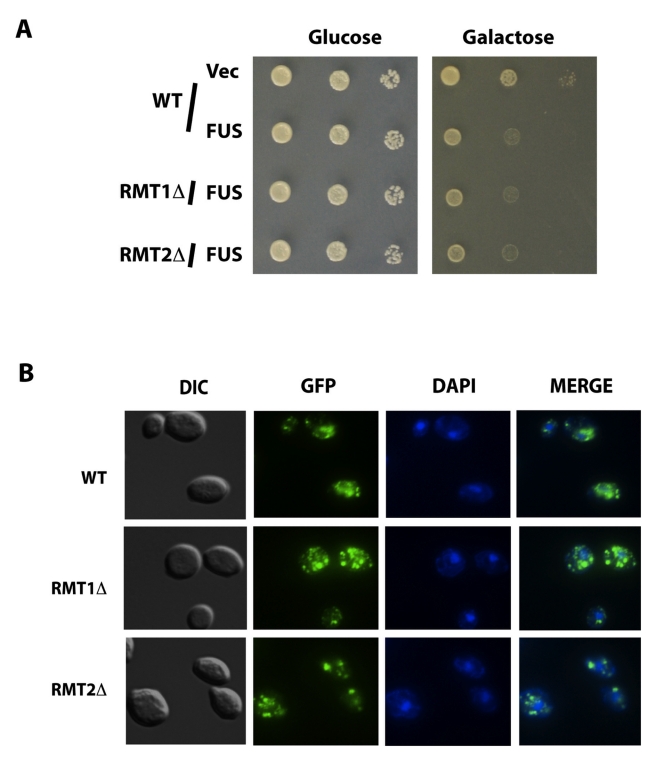
Deletion of arginine methyl transferase does *not* rescue FUS/TLS toxicity nor change its localization. (A) Empty vector and N-terminus GFP-tagged FUS/TLS on pYES2CT were transformed into yeast arginine methyl transferase deletion strain *rmt1Δ*, *rmt2Δ*, and its isogenic wild type BY4743 (WT). Spotting assay was performed to observe toxicity from the above yeast strains. (B) Expression of the proteins from above strains was induced by 2% galactose for 6 h. Localization and aggregation of the protein was visualized by fluorescence microscopy.

Most mutations of FUS/TLS identified in fALS patients are clustered at the very end of the C-terminus (residues 510–526), in a region enriched with arginine residues [5]. At least one disease-causing mutation has been identified for each arginine in this region, implying that these arginine residues play a critical role for the function of the protein. One regulatory process involving arginine residues is dimethylation, which is an important signal for the nuclear/cytoplasmic translocalization of a series of RNA binding proteins. That more than 20 arginine residues are indeed dimethylated by the enzyme PRMT1 in FUS/TLS in mammalian cells [32], and that all the known C-terminal region mutant forms of FUS/TLS do translocate to the cytosol, together suggest that arginine methylation may be involved in the shuttling of FUS/TLS between nucleus and cytosol and thus may play a role in its toxicity. To explore the possible role of the yeast arginine methyl transferases in mislocalization, localization and toxicity of FUS/TLS was studied in yeast strains in which each of the two major yeast arginine methyltransferases were deleted (*rmt1Δ* and *rmt2Δ*). As shown in Figure 4, FUS/TLS is still toxic in *rmt1Δ* and *rmt2Δ* strains (Figure 4A), and the protein is still aggregated in the cytosol in both cases (Figure 4B).

To test the possible redundancy of arginine methyltransferase activity in yeast, two small molecule compounds (AMI-1 and AMI-4), previously shown to exhibit broad inhibition of arginine methyl transferase activity in mammalian cells and in yeast [33], were tested on the yeast strain over-expressing FUS/TLS. Consistent with the results from the arginine methyl transferase deletion study, inhibition of arginine methyl transferase by these two compounds does not change FUS/TLS toxicity nor its localization (unpublished data). In addition, over-expression of *RMT1* and *RMT2*, or of the human enzyme PRMT1, does not modify FUS/TLS toxicity (unpublished data). These data suggest that at least the predominant yeast arginine methyl transferases are not involved in the cytotoxicity of FUS/TLS in yeast. We next examined the possible role of nuclear localization signals in the nuclear/cytosolic distribution of the human protein when expressed in yeast.

### The NLS of Human FUS/TLS Is Not Efficient in Yeast; However, Cytosolic Localization Is Correlated with Toxicity, Consistent with Findings for Neuronal Cells

**Figure 5:**
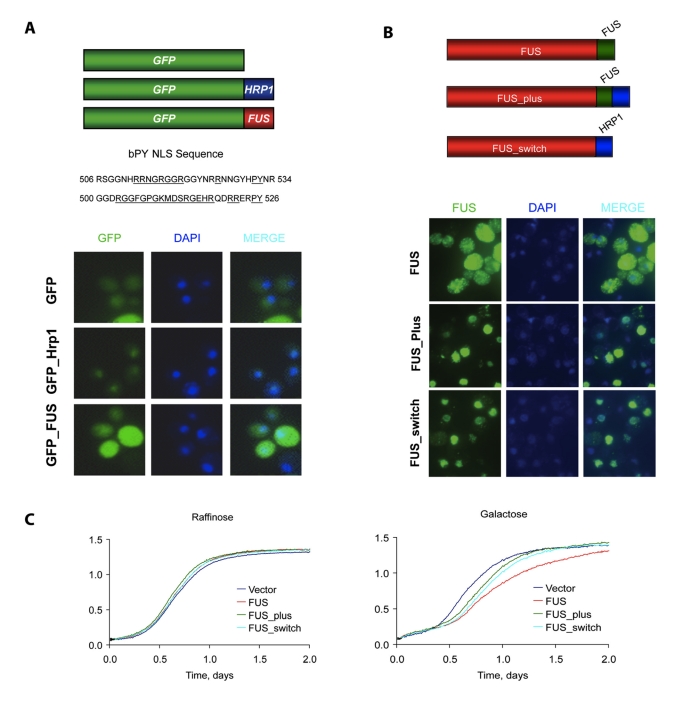
FUS_plus and FUS_switch promote nuclear localization and lower toxicity of FUS/TLS. (A) GFP was fused with *the* nuclear localization signal of FUS/TLS (GFP_FUS) and Hrp1 (GFP_Hrp1) as shown in top part of (A). Expression of protein from yeast containing above constructs was induced by 2% galactose for 6 h. Localization and aggregation of protein was visualized by fluorescence microscopy. (B) Hrp1 NLS was added to the c-terminal end of FUS/TLS (FUS_plus) or was used to replace nuclear localization signal of FUS/TLS (FUS_switch), as shown on part top of (B). Protein expression from yeast containing above constructs was induced by 0.1% galactose for 6 h. Protein localization and aggregation was visualized by fluorescence microscopy. (C) The same yeast strains were grown in raffinose medium and 0.1% galactose medium. Cell growth was monitored using a Bioscreen machine for 2 d at 30°C. Typically, at least 10 replicates were done for each.

It was recently reported that FUS/TLS carries a non-classical PY nuclear localization signal (NLS) in its extreme C-terminus (approx. residues 514–526) and this is necessary for its nuclear import [19]. The disease-causing mutations clustered in this NLS affect the nuclear localization of the protein. The toxicity of the protein and the age of disease onset correlate with the protein's cytosolic mislocalization and aggregation [19],[20],[31]. Since wild-type FUS was mostly aggregated in punctate granules in the yeast cytosol, we posited that its NLS might not be functional in this organism. If so, over-expression of even the wild-type protein would recapitulate the toxicity of the human mutants, as observed, through a failure in nuclear localization. To test this directly, we compared the ability of the FUS NLS to direct nuclear localization with a well-characterized yeast NLS that uses a similar PY sequence (from Hrp1) by fusing them to GFP. Indeed the FUS NLS was defective in nuclear localization (Figure 5A).

To determine if the failure in nuclear localization contributes to toxicity, we tested two constructs, one in which the HRP1 NLS was simply appended to the human protein (FUS_plus) and another in which the HRP1 NLS was used to replace the FUS/TLS (FUS_switch; Figure 5B). Both constructs dramatically increased nuclear localization of FUS (Figure 5B) and both reduced toxicity (Figure 5C). Aggregation was also reduced when FUS was retargeted to the nucleus, but this is probably due to a generally lower level of FUS expression in these constructs. Toxicity was not completely ameliorated, likely because some residual cytoplasmic FUS persisted even when augmented with the HRP1 NLS (Figure 5B). This relationship between mislocalization and toxicity is consistent with data from neuronal cells [19] and suggests that the wild-type protein is toxic in yeast because the nonfunctional NLS mimics the mislocalization effect of the disease-producing mutations in human cells.

### Genome Wide Screen to Identify Genes Whose Over-expression Rescues the Toxicity of FUS/TLS in Yeast

The ability of yeast expressing human FUS/TLS to recapitulate several salient features of disease prompted us to perform a genome-wide over-expression screen. By identifying yeast genes that modify FUS/TLS toxicity, we hoped to identify pathways or proteins that would illuminate pathogenic mechanisms. We screened an over-expression library containing a collection of yeast open reading frames, fully sequenced and placed under the control of a galactose-inducible promoter. A total of 5,535 genes are covered in this library (representing 95% of the yeast genome). We transformed each of the 5,535 genes into the yeast strain expressing the moderately toxic one copy of FUS/TLS (untagged FUS/TLS integrated into the yeast genome at the *HIS3* locus) and selected for those that suppress FUS/TLS toxicity upon over-expression. All of the positive over-expression plasmids were retransformed into a fresh yeast strain and validated for their suppressive effects. Surprisingly, after three rounds of retesting, only a handful of yeast genes could suppress FUS/TLS toxicity, all of which are listed in the Table 1. FUS expression is very toxic in yeast, and the identified suppressors do not completely abolish the effect of FUS on yeast growth. As observed for a similar screen in a yeast alpha-synuclein toxicity model [26], several transcription factors that down-regulate the GAL1 promoter activity also suppress the toxicity of FUS/TLS (Table 1, bottom section). These modifiers were not specific to FUS/TLS; they also suppress other toxic proteins expressed under GAL1 promoter control, such as TDP-43.

**Table 1:** Yeast genes rescuing the toxicity of human FUS/TLS when over-expressed.

Gene	Function	Human Homologue	Function of Human Homologue
**Genes suppressing FUS/TLS toxicity when over-expressed**			
ECM32	Member of the Dna2p- and Nam7p-like family of RNA helicases; involved in translation termination	UPF1	Nuclear mRNA export, mRNA surveillance, nonsense-mediated mRNA decay, Staufen1-mediated mRNA decay, replication-dependent histone mRNA decay, DNA synthesis and repair, telomere maintenance
NAM8*	RNA binding protein; component of the U1 snRNP protein complex involved in mRNA maturation	TRNAU1AP	Unknown; contains a putative RNA-binding domain
SBP1	Putative RNA binding protein; localizes to P-bodies and associates with snRNPs	RBM14	Nuclear receptor coactivator
SKO1	Transcription factor of the ATF/CREB family	None	
VHR1	Transcriptional activator	None	
**Genes regulating GAL1 promoter/general gene expression**			
MBP1	Transcription factor	None	
MIG1	Multicopy inhibitor of GAL gene expression	None	
MIG3	Transcriptional repressor		
REG1	Negative regulator of glucose-repressible genes	None	
ZDS1	Transcriptional silencing	None	
ZDS2	Transcriptional silencing	None	

*Identified in a number of other yeast suppressor screens. May affect GAL-driven gene expression.

**Figure 6:**
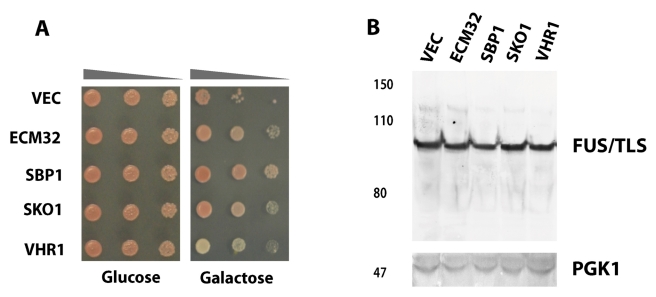
Expression of *ECM32*, *SBP1*, *SKO1*, and *VHR1* rescues FUS/TLS toxicity. (A) *ECM32*, *SBP1*, *SKO1*, and *VHR1* were individually transformed into 1X FUS strain. Spotting assay was performed to observe toxicity of yeast containing the above constructs. (B) Protein expression was induced by 2% galactose for 6 h from above yeast strains. Western blot analysis was performed using an antibody against FUS/TLS. PGK1 is shown as a control of protein loading.

All the FUS/TLS-specific suppressors are DNA/RNA binding proteins (Table 1, top section; and Figure 6A), including *ECM32*, *SBP1*, *SKO1*, and *VHR1*. As shown in Figure 6B, FUS/TLS protein level was not altered by over-expression of these four genetic modifiers, supporting the hypothesis that the rescue is not mediated by reducing the amount of FUS/TLS.

*ECM32* (also called *MTT1*) encodes a DNA-dependent ATPase/DNA helicase belonging to the Dna2p- and Nam7p-like family of helicases that are involved in modulating translation termination [34]. (Interestingly, we also detected *NAM8*, an RNA binding protein that interacts genetically with *NAM7* in yeast, as a suppressor of FUS toxicity. We include it on our list, but we are uncertain of its specificity; it appears as a suppressor in many screens and may cause some nonspecific downregulation of the GAL1 promoter. Yeast *NAM7*, which is sometimes called yeast *UPF1*, is a homologue of *ECM32*, but neither we nor Sun et al. [30] found *NAM7* as a strong suppressor in our screen.) Over-expression of ECM32 is known to induce a nonsense suppression phenotype in a wild-type yeast strain, and the ECM32 gene product has been shown to interact with translation termination factors and is localized to polysomes [34]. ECM32 is homologous to the human gene hUPF1, which encodes a protein previously shown to function in both mRNA turnover and translation termination, and which can be found in P-bodies, cytoplasmic granules that are sites of mRNA sequestration and turnover, including nonsense-mediated decay (NMD).

SBP1 encodes a putative RNA binding protein that is involved in translational repression and is also found in cytoplasmic P-bodies [35].

SKO1 encodes a basic leucine zipper transcription factor of the ATF/CREB family, which forms a complex with Tup1p and Ssn6p that acts as a repressor of transcription. In response to osmotic and oxidative stress, this complex can be converted into an activator that recruits SAGA and SWI/SNF [36].

VHR1 is a transcriptional activator that is required for the vitamin H–responsive element (VHRE) mediated induction of VHT1 (Vitamin H transporter) and BIO5 (biotin biosynthesis intermediate transporter) in response to low biotin concentrations [37]. In humans, biotin deficiency leads to a variety of clinical abnormalities, including neurological disorders, growth retardation, and dermal abnormalities [38].

All of these genes were identified by Sun et al. in an independent screen for suppressors in a similar yeast model for FUS/TLS-dependent proteotoxicity [30]. Differences between results of the two screens probably reflect differences in the protocol of the initial pass, plus differences in stringency in retesting.

**Figure 7:**
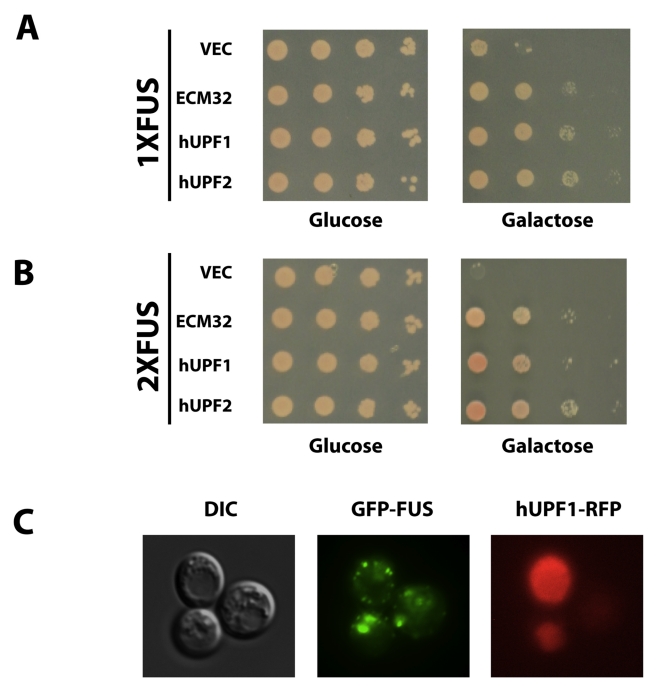
hUPF1 rescues FUS/TLS toxicity. *hUPF1* and *hUPF2* were cloned into yeast expression vector pYES2CT. (A) The constructs were transformed into 1XFUS (one copy of FUS/TLS integrated at HIS locus). Spotting assay was performed to check the rescue of toxicity by *hUPF1* and *hUPF2.* Empty vector and *ECM32* construct from library screen were used as negative and positive controls. (B) The above constructs were transformed into 2XFUS (two copies of FUS/TLS integrated at HIS locus and TRP locus, respectively). Spotting assay was performed to check the rescue of toxicity by *hUPF1* and *hUPF2.* (C) GFP tagged FUS/TLS (pYES2CT/GFP-FUS) and RFP tagged hUPF1 (pRSGal1hUPF1-DsRed) were transformed into yeast. Protein expression was induced by 2% galactose for 6 h and visualized by fluorescence microcopy.

Of the genetic modifiers identified from our screen, *ECM32* is the only gene that is capable of rescuing toxicity of yeast strains integrated with both one copy (Figure 7A) and two copies of FUS (Figure 7B). The other suppressors only rescue toxicity of 1XFUS (unpublished data). We therefore turned our attention to the human homologues of this protein.

### hUPF1, a Human Homolog of *ECM32*, Rescues Toxicity of FUS/TLS

Based on sequence similarity (∼30% identity and ∼50% similarity in the helicase domain), *hUPF1*, a gene playing an important role in the pathway of nonsense-mediated decay (NMD), is the closest human homolog of *ECM32* [39]. Because of this homology, we hypothesized that the toxicity of FUS/TLS may be rescued by yeast expression of *hUPF1*. The full-length *hUPF1* gene was cloned into a yeast expression vector and tested on the toxicity of FUS/TLS. Over-expression of *hUPF1* rescues the toxicity of both 1XFUS and 2XFUS (Figure 7A and B). To check for possible direct interaction between hUPF1 and FUS/TLS, we co-expressed red fluorescent protein-tagged hUPF1 and GFP-tagged FUS/TLS. hUPF1 is expressed mainly in the cytosol in yeast but does not co-localize with FUS/TLS (Figure 7C), suggesting the rescue effect by hUPF1 might be indirect. However, more data are needed to rule out possible over-expression artifacts.

Next, we investigated whether the expression of *hUPF2*, another nonsense-mediated decay pathway gene whose protein product is known to form a complex with the UPF1 protein, might also rescue FUS toxicity in yeast, and found that it did, to an equal extent as that of *hUPF1* (Figure 7). However, over-expression of human *UPF3*, another protein known to interact with UPF1, showed only moderate rescue compared to *hUPF1* and *hUPF2* (suppression of toxicity of 1XFUS but not of 2XFUS). We then examined the expression of the next closest human homologue of *ECM32*, *IGHMBP2* (∼25% identity), a ribosome-associated helicase implicated in DNA replication, pre-mRNA splicing, and transcription [40]. Mutations in *IGHMBP2* cause distal spinal muscular atrophy type 1, a neuromuscular disorder [40]. However, expression of *hIGHMBP2* in yeast did not rescue FUS/TLS toxicity (unpublished data).

**Figure 8:**
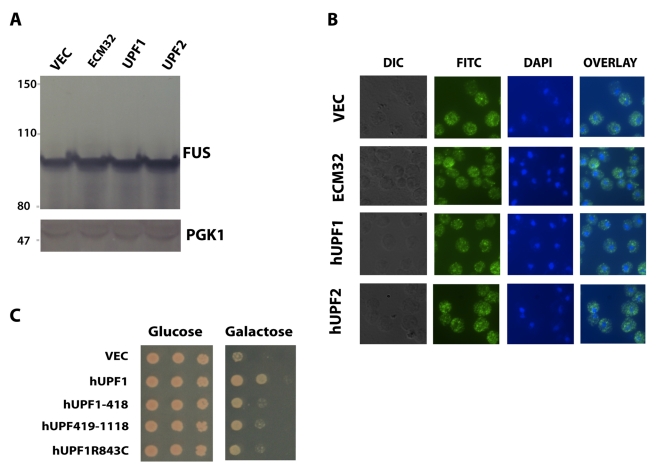
Rescue of FUS/TLS toxicity requires full length *hUPF1*, and rescue is *not* mediated by decrease in FUS/TLS protein level or inclusion formation of the protein. (A) Protein expression was induced by 2% galactose for 6 h in 1XFUS strain expressing hUPF1, hUPF2, or ECM32. Western blot was performed using an antibody against FUS/TLS to check the expression of FUS/TLS. PGK1 is shown as a control of protein loading; (B) the same yeast cells were also subject to indirect immunofluorescence staining using primary antibody against FUS/TLS, and secondary antibody conjugated with fluorescein. Nuclear DNA was stained with DAPI. (C) Different domains of *hUPF1* were cloned into a yeast expression vector and transformed into 1XFUS strain. Spotting assay was performed to check rescue of the toxicity. hUPF1-418, hUPF2 binding domain; hUPF419-1118, ATPase/Helicase domain; hUPF1R843C, inactivated ATPase/Helicase by arginine to cysteine mutation at residue of 843 in the full length FUS/TLS.

To check the possible effect of *ECM32*, *hUPF1*, and *hUPF2* on the protein expression level or aggregation of FUS/TLS, Western blots and indirect immunofluorescence using FUS/TLS antibody were performed. Neither protein levels (Figure 8A) nor localization or aggregation of FUS/TLS (Figure 8B) was modified by over-expression of *ECM32*, *hUPF1*, or *hUPF2*.

hUPF1 has an N-terminal hUPF2 binding domain and a C-terminal ATPase/Helicase domain. To determine whether the rescue of FUS/TLS toxicity requires both domains, we tested the constructs expressing only the hUPF2 binding domain (hUPF1-418) or the ATPase/Helicase domain (hUPF419-1118). As shown in Figure 8C, neither domain rescues the toxicity of FUS/TLS to the extent that the full-length wild type protein rescues. To further test whether ATPase/Helicase activity is required for the rescue, we checked the full-length protein with ATPase/Helicase partially inactivated by a point mutation (R844C) [41] and found that FUS/TLS toxicity cannot be fully rescued when ATPase/Helicase activity of hUPF1 is inhibited. It is noteworthy that hUPF1(R844C) still has 60% of the activity of wild-type hUPF1 [41], so it is quite possible that fully inactivated hUPF1 would not rescue at all. These data suggest that both domains of hUPF1 and functional ATPase/Helicase activity are required for its rescue of FUS/TLS toxicity.

### CYH2 But Not MER2 Pre-mRNA Was Accumulated When FUS Was Over-expressed

**Figure 9:**
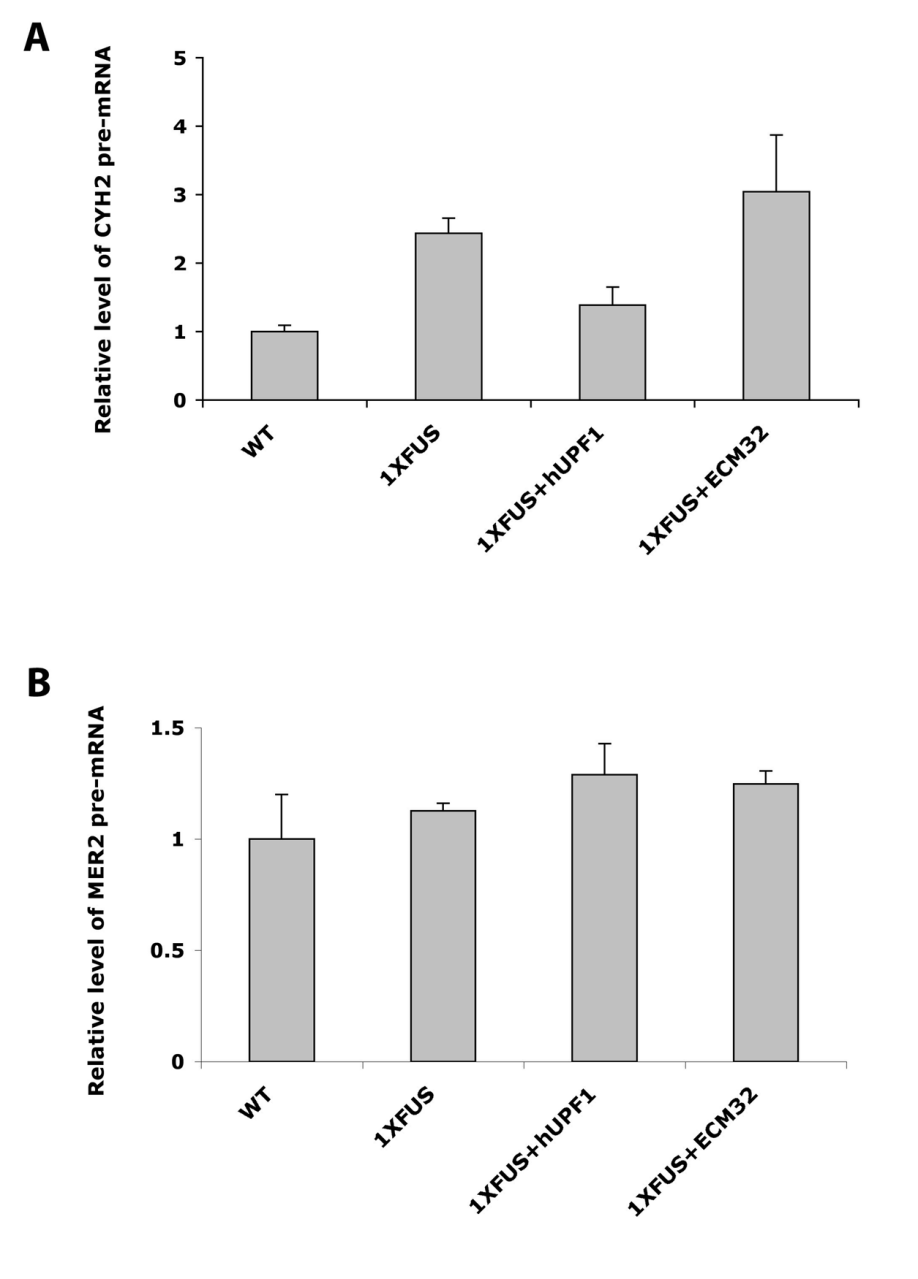
Yeast *CYH2* but not *MER2* pre-mRNA was accumulated when FUS is over-expressed. Cells were grown in raffinose medium to early log phase. Protein expression was induced by 2% galactose for 6 h in 1XFUS strain expressing empty vector (1XFUS), human UPF1 (+hUPF1), or *ECM32* (+ECM32). CYH2 pre-mRNA (A) and MER2 pre-mRNA level (B) were determined by qRT-PCR using 18sRNA as an internal control. Pre-mRNA in wild type yeast cell without integration of FUS (WT) was normalized to 1.

To prevent the potential accumulation of deleterious nonsense fragments of polypeptides in the cytoplasm, mRNAs that retain an intron containing an in-frame nonsense codon are usually degraded by the nonsense-mediated decay (NMD) pathway. It is long established that *CYH2* and *MER2* pre-mRNA are among the substrates of this pathway in yeast. These pre-mRNAs are accumulated 2- to 5-fold when the NMD pathway is deficient [42]. To check the potential effects of FUS expression on the NMD pathway, qRT-PCR was utilized to determine *CYH2* and *MER2* pre-mRNA levels in 1XFUS yeast, and in its suppressor strains. As shown in Figure 9A, *CYH2* pre-mRNA was increased about 2-fold when FUS is over-expressed, and co-expression of its suppressor hUPF1 brought *CYH2* pre-mRNA back to the wild type level; however, co-expression of another suppressor, *ECM32*, did not, suggesting that these two suppressors may rescue FUS toxicity through different mechanisms.

In contrast to *CYH2*, over-expression of FUS and co-expression of its suppressors did not change *MER2* pre-mRNA levels (Figure 9B). Since *MER2* pre-mRNA is another substrate of the NMD pathway, this result implies that accumulation of *CYH2* pre-mRNA by FUS over-expression is not through its direct effect on the NMD pathway but through an effect on one or more additional pathways of mRNA quality control. Possibly, the restoration of the level of *CYH2* pre-mRNA to normal by hUPF1 expression in the 1XFUS strain also does not reflect hUPF1 function in NMD.

## Discussion

Many essential cellular functions are conserved in the simple eukaryote yeast. Studies from this organism have provided valuable information for our understanding of many critical cellular functions, including cell cycle regulation, DNA replication, RNA synthesis and processing, protein synthesis, protein trafficking, and signal transduction. This simple system has also been utilized to study functions of proteins involved in human diseases, including neurodegenerative diseases. Although a yeast model usually cannot recapitulate all of the cellular processes in human cells, it has proven to capture key aspects of molecular pathology for several neurodegenerative disorders [27]. With its ease of high-throughput manipulations for both genetics and biochemistry, the yeast model organism provides invaluable tools for studying molecular mechanisms of human diseases. For those human proteins for which yeast cytotoxicity models are available, toxicity from each protein is usually quite different. Genetic modifiers identified from those yeast models usually do not overlap, supporting the use of yeast models for studying functions of human proteins specifically.

In this article, we report a yeast model of FUS/TLS-associated proteotoxicity when the protein is mislocalized to the cytoplasm. The model faithfully recapitulates the cytosolic aggregation and cytotoxicity observed in spinal motor neurons in the human disease. We exploited this model to test various hypotheses about FUS-mediated cytotoxicity. Comparison of the aggregates isolated from the model with those from yeast models of huntingtin toxicity and TDP-43 toxicity showed that they differed from the former but were similar to the latter; two yeast genes known to affect huntingtin aggregation when over-expressed also failed to affect FUS/TLS localization or aggregation. Clustering of fALS-associated FUS mutations in regions of potential arginine dimethylation prompted us to investigate the effects of deletion of either of the major yeast arginine methyltransferases on FUS/TLS cytosolic localization and toxicity, but neither their deletions nor introduction of known chemical inhibitors of yeast arginine methyltransferase activity had any effect on these properties. Over-expression of the major yeast and human arginine methyltransferaaes also failed to modulate FUS/TLS toxicity in yeast. However, we have no data at present to indicate that human FUS/TLS is a substrate for the yeast arginine methyltransferases (even though they are close homologues of the major human arginine methyltransferase, PRMT1), so we cannot conclude from this experiment that arginine methylation may play no role in FUS/TLS toxicity in mammalian cells. It does not seem to be a major factor in toxicity in yeast.

Initially, the observation that both WT and mutant FUS localized to the cytoplasm and were equally toxic was unexpected. However, recent work on the C-terminal FUS mutations provides a satisfying explanation [19]. These mutations prevent the nuclear import of FUS, increasing cytoplasmic accumulation in stress granules and, eventually, producing toxic and insoluble aggregates. FUS uses an unusual nuclear localization signal (NLS) of the bPY-type. Although yeast has this same bPY-type nuclear localization system, divergence in the recognition signal would cause toxic mislocalization of even the WT FUS protein in yeast. Indeed, when we compared the ability of the WT FUS NLS signal and a known functional yeast bPY-type NLS to drive a GFP reporter into the nucleus, the FUS signal was nonfunctional. Next, we reasoned that if cytoplasmic mislocalization of FUS was responsible for increased toxicity, then restoring nuclear localization with a recognition sequence that does function in yeast should reduce toxicity. This proved to be correct. Thus, our work provides an independent validation of Haass' recent model [19], recapitulating the observation that cytoplasmic mislocalization is important in the toxicity of FUS. This mechanistic link between mutations and toxicity is in contrast to TDP-43, where ALS mutations increase aggregation in vitro (as opposed to transport) and enhance toxicity in yeast [28]. In agreement, the accompanying manuscript demonstrates that FUS mutations do not alter aggregation or toxicity [30]. The lack of an effect of ALS mutations in yeast separates two aspects of FUS pathology—(1) mislocalization and (2) cytoplasmic toxicity. Since the NLS is nonfunctional in yeast, our system models the cytoplasmic-dependent toxicity, but not the mechanism of mislocalization itself. Because the link between mislocalization and ALS mutants has been established, we view the inability to model mislocalization not as a liability but rather as a strength, in that it allows us to focus on toxicity itself.

We also expressed a series of FUS/TLS constructs with various domains deleted and found that the C-terminal region of the protein was necessary but not sufficient for toxicity. In an independent, more detailed study using a similar yeast model of FUS/TLS toxicity, Sun et al. [30] conclude that, in contrast to TDP-43, determinants in both the N- and C-terminal regions of FUS are required to couple aggregation to toxicity in vivo and for spontaneous aggregation in vitro, suggesting that FUS aggregates by a mechanism distinct from that of TDP-43. They also find that FUS is intrinsically aggregation-prone and that the aggregates formed by purified FUS in vitro closely resemble the aggregates observed in affected neurons in human disease.

Some of the aggregates formed by the various deletion constructs appeared to differ from one another slightly when viewed microscopically. We have not yet characterized the nature of the aggregation in any of these cases, but if different morphological aggregates are indeed toxic and similar-looking aggregates are not always toxic, as we in fact observe, our data lend support to the conclusion that aggregation and toxicity may not be tightly coupled in this system.

Using a yeast over-expression library screen, we identified five yeast genes that, when over-expressed, rescue the toxicity of FUS/TLS. Strikingly, all five genetic modifiers are, like FUS, DNA/RNA binding proteins. We have compared genetic modifiers from other yeast models and found that these five genes are not identified as suppressors in other yeast models for neurodegenerative diseases, including Parkinson's disease (over-expression of α-synuclein [25]), TDP-43-dependent ALS (over-expression of wild-type TDP-43 [28]), and Huntington's disease (over-expression of polyQ-expanded huntingtin [24]), indicating that they are specific to FUS/TLS. We also note that genetic screens from other yeast models usually identify many more genetic modifiers. The very limited number of hits from our FUS/TLS yeast model suggests that the toxicity of FUS/TLS may stem from its effect on a limited number of cellular functions. Our screening results are similar to results obtained independently by Sun et al. [30], who found 23 over-expression suppressors (including all of the genes we identified) and also carried out a screen for yeast genes that modify toxicity when deleted. Further, they provide compelling evidence that stress granules and P-bodies are likely to be involved in FUS/TLS effects in yeast. We have also found a correlation between mutant FUS/TLS and stress granules that may be relevant to ALS pathogenesis [20].

Most importantly, we found that expression of *hUPF1* (or of its physical interacting partner *hUPF2* and, to a lesser extent, h*UPF3*) rescues FUS/TLS toxicity. Among other roles, *hUPF1* plays a very important function in mRNA quality control, including nonsense-mediated decay (NMD), a critical cellular mechanism of mRNA surveillance that functions to detect nonsense mutations and prevent the expression of truncated or erroneous proteins [43]. It has been proposed that a principal event underlying neurodegeneration occurs when cytotoxic, truncated proteins are expressed from normally degraded nonsense-containing RNAs and pseudogene transcripts [44]. Our finding that *hUPF1* and *hUPF2* rescue the toxicity of FUS/TLS is broadly consistent with this hypothesis; however, our results from examination of the level of specific NMD mRNA substrates (see below) suggest that NMD cannot be the sole RNA pathway affected by FUS or its suppressors in yeast. Our results do suggest the possibility that disruption of some part of the RNA quality control process might be related to the toxicity mechanism of FUS/TLS.

Because *ECM32*, *hUPF1*, or *hUPF2* expression all rescue FUS toxicity without dissolving the cytosolic aggregates or changing the expression level of FUS or its mislocalization, it is likely that toxicity involves disruption of some essential cellular function that is either restored or compensated for by the introduction of these genes. One possibility is that FUS over-expression sequesters RNA and/or protein molecules involved in nonsense-mediated decay, which is an essential function in yeast. Yeast contains no FUS homologue, but many of the other proteins important for RNA quality control are conserved between *S. cerevisiae* and humans.

To check the possible direct effect of FUS on the NMD pathway, we determined pre-mRNA levels of *CYH2* and *MER2*, which are among the reported substrates of the NMD pathway in yeast. If this pathway is impaired by FUS over-expression, it is then expected that both pre-mRNAs would be accumulated. However, only *CYH2* pre-mRNA is increased by FUS expression in our assay, suggesting that FUS may interfere with other RNA quality control systems, rather than exerting a direct effect on the NMD pathway. It is worth mentioning that *CYH2* pre-mRNA was accumulated to a much higher level than *MER2* in our assay (5-fold versus 2-fold; unpublished data); this may help to explain no detected accumulation of *MER2* pre-mRNA when FUS is over-expressed.

In addition, co-expression of hUPF1 and yeast *ECM32*, two suppressors of FUS toxicity, had different effects on the accumulated *CYH2* pre-mRNA level caused by over-expression of FUS. Together with their different rescuing effects on other yeast neurodegenerative disease models, these data suggest that hUPF1 and *ECM32* may rescue FUS toxicity through different mechanisms.

It is important to emphasize that we are not claiming that this is a yeast model of a human disease. It is a model for the cytotoxicity of a human protein whose mislocalization to the cytosol causes a devastating neurologic disorder. It appears to recreate the salient features of that part of the pathology: cytosolic localization, aggregation in stress granules, and cell death. It has allowed us to determine the parts of the protein essential for toxicity, to test hypotheses about the factors responsible for localization, and to identify suppressor genes in both the yeast and human genome. We believe that the fact that wild type and mutant are both toxic in this model is not a failing of the model. Both wild-type and mutant are mislocalized to the same extent in yeast because the FUS nuclear localization signal, where the mutations occur, is not efficient in the microbe, and so if the neurotoxicity of the mutants is entirely due to their mislocalization, as has been hypothesized by others, then the wild type protein *should* also be toxic in our model, exactly as observed.

In summary, our yeast model recapitulates multiple features of disease-causing mutant protein FUS/TLS pathology, including aggregation, cytosolic localization, and toxicity, which should make it valuable for studying the function and mechanism of toxicity of this protein in human neurodegenerative disorders. In addition, our model is amenable to high-throughput small molecule screens to identify compounds that suppress FUS/TLS toxicity. Like TDP-43, which saw a number of cell culture and animal models follow its identification as an ALS/FTLD protein, we envisage a similar trajectory for FUS/TLS. By importing our yeast findings into mammalian cell culture and neuronal systems, we anticipate creating a yeast discovery/mammalian confirmation paradigm that will yield critical insights into FUS/TLS pathobiology and potentially provide therapeutic targets or pathways for exploitation.

## Materials and Methods

### Plasmids

N-terminal GFP-tagged FUS (pYES2/GFP-FUS): GFP tagged FUS gene was amplified from pDEST53/FUS by PCR using forward primer 5′- ATTAGCCGGGTACCATGGCCTCAAACGATTATACCC-3′, and reverse primer 5′-ATTAGCCGTCTAGATTAATACGGCCTCTCCCTGC-3′, and sub-cloned into KpnI and XbaI sites of yeast expression vector pYES2CT (Invitrogen).

Entry clone of FUS (pDONR221/FUS): full-length FUS gene in destination vector pDEST53/FUS was transferred into Gateway entry vector pDONR221 (Invitrogen) using BP reaction (Invitrogen).

Yeast expression and integration constructs of FUS: A gateway LR reaction (Invitrogen) was used to shuttle FUS gene from entry clone into gateway compatible yeast expression vectors (pAG vectors, www.addgene.org/yeast_gateway).

Yeast expression vectors of UPF1 (pYES2/UPF1) and UPF2 (pYES2/UPF2): UPF1 and UPF2 were amplified by PCR. The genes were generously supplied by Dr. Lynne Maquat of Rochester University School of Medicine and Dentistry. UPF1 was sub-cloned into BamHI and XhoI sites of pYES2CT, and UPF2 was sub-cloned into NotI and XhoI sites of pYES2CT.

GFP and FUS NLS fusion constructs were generated using an overlap PCR strategy. GFP and HRP1 or FUS/TLS NLSs were PCR amplified in the first step. In the second step, GFP was combined with either HRP1 or FUS/TLS NLS PCR using the GFP forward primer and HRP1 or FUS/TLS reverse primer. This product was cloned into Pst1/Spe1 sites in the pRS424Gal1 vector. For “FUS_plus” or “FUS_switch” constructs, the first PCR step amplified FUS or FUS lacking the C-terminal PY NLS and the Hrp1 NLS. Full-length FUS or NLS-lacking FUS were combined with the Hrp1 NLS PCR product and amplified in a second reaction containing a FUS forward primer and HRP1 reverse primer. This final product was cloned into pRS424Gal1. Oligo sequences are available upon request.

All constructs made for this study were confirmed by sequencing.

### Yeast Strains, Media, and Growth Conditions

1XFUS integration strain was generated by linearizing pAG303GAL1FUS with NheI, and followed by transformation into W303α strain (MATα can1-100, his3-11,15, leu2-3,112, trp1-1, ura3-1, ade2-1).

2XFUS integration strain was generated by linearizing pAG303GAL1FUS with NheI, and pAG304GAL1FUS with BstZ17I, followed by transformation into W303α strain (MATα can1-100, his3-11,15, leu2-3,112, trp1-1, ura3-1, ade2-1). Both 1XFUS and 2XFUS strains were confirmed by PCR.

Htt25 and Htt103 strains: N-terminal fragments of huntingtin with 23 glutamine repeats or 103 glutamine repeats, respectively, were integrated into HIS locus of W303 strain.

rmt1Δ, rmt2Δ, hsp104Δ, and rnq1Δ strains are homozygous diploid from the yeast deletion collection (Research Genetics). BY4743 is its isogenic wild type.

Synthetic media lacking uracil (Ura-), histidine (His-), histidine and tryptophan (His-Trp-), histidine and uracil (His-Ura-), and containing 2% glucose, raffinose, or galactose were used for the respective yeast strains.

Yeast cells were grown in 30° incubators (plate) or 30° shakers (liquid medium) unless specially mentioned.

Growth curves of FUS NLS strains were monitored using Bioscreen (www.bioscreen.fi). Yeast strains were pre-grown in 2% raffinose, diluted to an OD600 of 0.01, and induced with 0.1% galactose for 2 d with OD measurements taken every 10 min. Raw data were averaged among triplicates and OD600 plotted over time. Three independent experiments were performed and a representative shown.

### Yeast Over-expression Library

The over-expression library is the FLEXGene Collection [45]. Additional information about the yeast FLEXGene Collection is available at http://plasmid.med.harvard.edu/PLASMID/GetCollection.do?collectionName=HIP%20FLEXGene%20Saccharomyces%20cerevisiae%20%28yeast%29%20ORF%20collection%20%28pBY011%20expression%20vector%29. For the expression screen, the clones were transferred into a galactose-inducible expression plasmid (pBY011; *CEN*, *URA3*, Amp^R^) using the Gateway technology (Invitrogen).

### Yeast Transformation

Yeast expression constructs were transformed using standard PEG/lithium acetate method. Briefly, cells from one-milliliter overnight culture plus DNA construct was mixed with transformation buffer (80 µl 50% PEG3350, 10 µl 1M DTT, and 10 µl 2M LiAC), followed by incubation at 42° waterbath for 45 min (with occasional mix during the incubation). Cells were then spread onto respective dropout plates and grown at 30° for 3–4 d.

### Serial Dilution and Spotting

Yeast cells were grown overnight to mid-log phase. Cultures were then normalized to OD600  = 5.0, and 10× serially diluted and spotted onto the respective dropout plates containing 2% glucose or galactose.

### Immunobotting

Yeast crude extract was subjected SDS-PAGE, and protein was transferred onto PVDF membrane (Millipore), followed by 30 min incubation with superblock (Thermosci). PVDF membrane was then hybridized with primary antibody for 2 h at RT, followed by wash with 1XPBS 5 times (10 min each), incubation with secondary antibody conjugated with alkaline phosphatase (Promega) for 2 h, and 5×10 min wash with 1XPBS. The membrane was finally developed with one-step NBT/BCIP solution (Thermo scientific). The anti-FUS antibody (Abcam) and anti-PGK1 (Invitrogen) were used at a dilution of 1∶1,000. The AP conjugated secondary antibody was used at dilution of 1∶10,000. For FUS NLS fusion experiments, cells were induced with 0.1% galactose for 6 h, after which they were fixed and visualized with anti-FUS antibody as described above.

### Fluorescence Microcopy of GFP-Tagged Protein

Cells were grown in selective raffinose medium to early log phase, and 2% galactose was then added into the medium for 6 h to induce the expression of the protein. Cells were harvested and fixed 1 h on ice in freshly made fixation buffer (50 mM Kpi pH 6.5; 1 mM MgCl2, and 4% formaldehyde). Cells were then washed 3 times with 1XPBS before viewing by fluorescence microscopy.

To visualize the nucleus, following the PBS washes, cells were incubated in PBS containing DAPI (1∶1,000) for 30 min. Cells were finally washed 3 times with 1XPBS before viewing by fluorescence microscopy.

### Filter Retardation Assay

Yeast cells were grown in raffinose medium to early log phase. Expression of protein was induced for 6 h by adding 2% galactose into the medium. Cells were harvested and treated with zymolase, and the spheroblast was broken by vortex, and protein extract was prepared by collecting the supernatant (centrifuge 5,000 rpm, 5 min). Protein concentration was determined by Bradford assay. 2% of SDS was added to the protein sample before the sample was boiled for 5 min. 10-fold dilutions of protein samples was prepared in 96-well plates, and loaded onto the prepared manifold (V&P scientific) with cellulose acetate membrane (pore size 2 µ; Whatman). Vacuum was applied and all the liquid was sucked through the manifold. After washing 5 times with 0.2% SDS, the manifold was dissembled carefully, and cellulose acetated membrane was used for Western blotting to detect protein.

### Indirect Immunofluorescence of Yeast Cells

Indirect immunofluorescence of yeast cells with FUS antibody was adapted from chapter 40 of “Guide to Yeast Genetics and Molecular Biology.” Yeast cells were grown to early log phase in the selective raffinose medium, and expression of the interested protein was induced for 6 h by 2% galactose. Cells were fixed in freshly made fixation buffer (50 mM Kpi pH 6.5, 1 mM MgCl2, and 4% formaldehyde) for 2 h at room temperature. Cells were then washed two times with PM buffer (0.1 M Kpi pH 7.5, 1 mM MgCl2) and resuspended with PM buffer with protease inhibitors (Roche). Cells were then treated with zymolase for 20 min. Spheroblasts were harvested at 2,000 rpm and washed once with PM with protease inhibitors. Cells were then spotted onto poly-l-lysine coated well of the slide. We immersed the slide for 5 min each in methanol and acetone (pre-cooled to −20°C). Cells were then blocked by PBS-block (1XPBS, 1% dried milk, 0.1%BSA, 0.1% octyl glucoside) for 1 h, followed by incubation with primary antibody (Abcam, 1∶100 dilution), wash, incubation with secondary antibody conjugated with fluorescein (Invitrogen, 1∶100 dilution), and wash.

5 µl of mounting solution (Santa Cruz Biotech) was added to each well, and cells were viewed using fluorescence microscope. To visualize nucleus, DAPI (1∶1,000) was included in the mounting solution.

### Yeast Over-expression Library Screen

One copy integrated FUS strain (1XFUS) was grown to early log phase and washed with 0.1 M lithium acetate (LiAc) in TE buffer. Cells were then resuspended in 0.1 M LiAC, and 35 µl of the resuspended cells was aliquoted into 96-well plates and incubated at 30° for 30 min. 1 µl of yeast FLEXGene Collection DNA (the Collection consists of vectors expressing each of 5,535 individual yeast genes, arrayed on 96-well plates; see [44]), and 125 µl transformation buffer (0.1 M Lithium Acetate, 10% DMSO, 40% PEG3350) was then added to the plate, followed by 30 min incubation at 30°, and 20 min heat shock at 42°. Cells were pelleted and resuspended into 200 µl of synthetic Ura- dropout medium, 10 µl of which was then inoculated into new plate with 200 µl Ura- dropout medium in each well. Cells were grown at 30°C for 2–3 d. All the liquid handling was done using liquid handling robot (Tecan Freedom EVO).

Cells were mixed using 96-well plate vortexer (VWR), and quadruply spotted onto Ura-Glucose and Ura-Galactose plates using Singer RoToR Robot (Singer Instruments), followed by incubation at 30° for 2–3 d. Colonies grown on galactose plates were considered as putative suppressors.

After the whole library (5,535 genes) was screened, all the putative suppressors were re-tested by re-transforming the corresponding genes into 1XFUS strain. Those surviving the re-test are finally confirmed by manually transforming each of the corresponding genes into 1XFUS stain, and phenotype was re-tested by serial dilution.

### Pre-mRNA Analysis by qRT-PCR

Cells were grown in synthetic raffinose medium to early log phase; expression of FUS and its suppressors (hUPF1 and *ECM32*) were induced by 2% galactose for 6 h. Cells were harvested, and total RNA was extracted using the standard hot acidic phenol method (Current Protocols in Molecular Biology, Unit 13.12). RNA was treated with DNase I (Promega) to remove the trace contamination of genomic DNA before it was used for cDNA synthesis. cDNA was synthesized using the superscript III platinum two-step qRP-PCR kit (Invitrogen). qPCR was performed on stepOnePlus real-time PCR system (Applied Biosystems). The PCR mixture contained platinum Taq and SYBR Green I (Invitrogen) and the corresponding primers: CYH2Pre forward: 5′-GTATCAAATGGTTGTAGAGAGCGC-3′, CYH2Pre reverse: 5′-TGTGGAAGTATCTCATACCAACC-3′; MER2Pre forward: 5′- GAACAAGATGCTGCTACGAACGGT-3′, MER2Pre reverse: 5′- TGCCTGTAGCTGGAATCCGACTTT-3′. mRNA levels were quantified and normalized to that of 18sRNA, using primers: 18sRNA forward: 5′- TTCTGGCTAACCTTGAGTCC-3′, and 18sRNA reverse 5′- AAA ACG TCC TTG GCA AAT GC-3′.

## Supporting Information

Figure S1 Inclusion formation and toxicity of FUS/TLS in wild type and mutant forms (H517Q and R521G) are comparable. (A) Cells expressing GFP-FUS on pYES2CT vector in both wild type and mutant forms (H517Q and R521G) were induced by 2% galactose for 6 h. Cells were then fixed and viewed by fluorescence microscopy. DAPI was used to stain the nucleus. (B) The same cells were subjected to Western blot analysis using an antibody against FUS/TLS. PGK1 is shown as a control of protein loading. (C) The spotting assay was performed to observe toxicity from the same yeast strains as above.
(TIF)

## Abbreviations

ALS: amyotrophic lateral sclerosis
fALS: familial amyotrophic lateral sclerosis
FUS: fused in sarcoma
GFP: green fluorescent protein
mRNA: messenger RNA
NIFID: neuronal intermediate filament inclusion disease
NLS: nuclear localization signal
NMD: nonsense-mediated decay
OPTN: optineurin
sALS: sporadic ALS
SETX: senataxin
TLS: translocated in liposarcoma
VAPB: vesicle associated membrane protein B
VHRE: vitamin H–responsive element
