# Supplementary figures and images for: A Yeast Model of FUS/TLS-Dependent Cytotoxicity
Source: PLoS Biol. 2011 Apr 26;9(4):e1001052. doi: 10.1371/journal.pbio.1001052 (PMC3082520; doi:10.1371/journal.pbio.1001052)

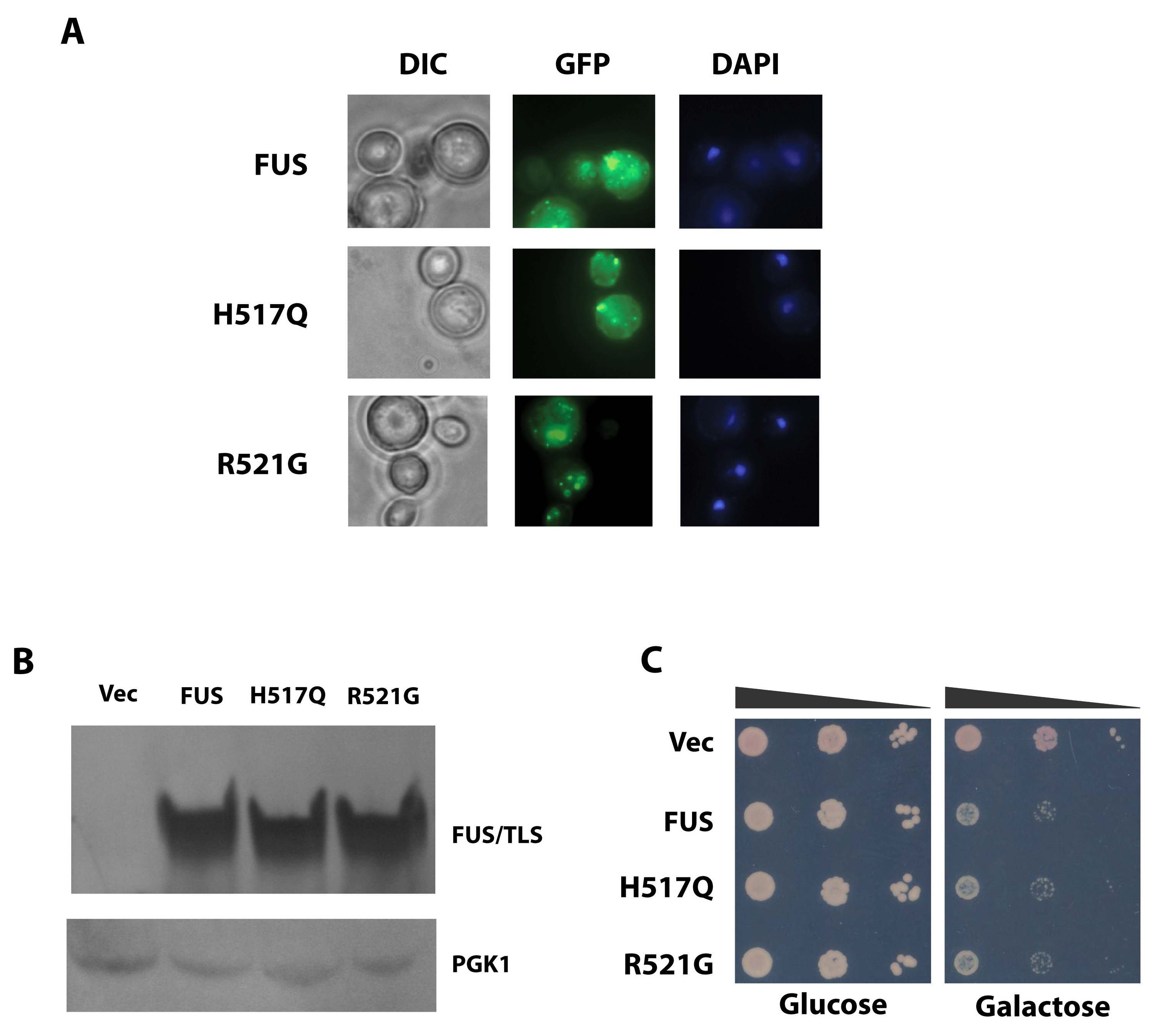

Supplement: Figure S1 — Inclusion formation and toxicity of FUS/TLS in wild type and mutant forms (H517Q and R521G) are comparable. (A) Cells expressing GFP-FUS on pYES2CT vector in both wild type and mutant forms (H517Q and R521G) were induced by 2% galactose for 6 h. Cells were then fixed and viewed by fluorescence microscopy. DAPI was used to stain the nucleus. (B) The same cells were subjected to Western blot analysis using an antibody against FUS/TLS. PGK1 is shown as a control of protein loading. (C) The spotting assay was performed to observe toxicity from the same yeast strains as above. (TIF) [file pbio.1001052.s001.tif]
